# Sandwich Immuno-RCA Assay with Single Molecule Counting Readout: The Importance of Biointerface Design

**DOI:** 10.1021/acsami.3c18304

**Published:** 2024-03-26

**Authors:** Katharina Schmidt, Tomas Riedel, Andres de los Santos Pereira, N. Scott Lynn, Diego Fernando Dorado Daza, Jakub Dostalek

**Affiliations:** † Laboratory for Life Sciences and Technology (LiST), Danube Private University, Viktor-Kaplan-Straße 2, 2700 Wiener, Neustadt, Austria; ‡ Institute of Macromolecular Chemistry, Czech Academy of Sciences, Heyrovského nám. 2, Prague 162 00, Czech Republic; § FZU-Institute of Physics, Czech Academy of Sciences, Na Slovance 2, Prague 182 21, Czech Republic

**Keywords:** rolling circle amplification, biomarker, surface plasmon resonance, surface plasmon-enhanced fluorescence, antifouling biointerface, single molecule detection, digital readout of assay

## Abstract

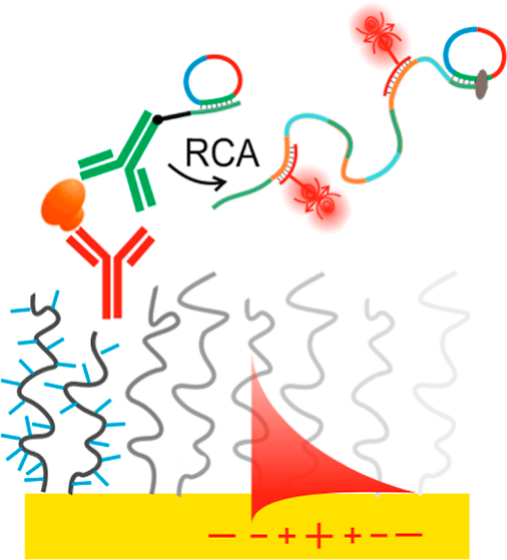

The analysis of low-abundance protein molecules in human serum is reported based on counting of the individual affinity-captured analyte on a solid sensor surface, yielding a readout format similar to digital assays. In this approach, a sandwich immunoassay with rolling circle amplification (RCA) is used for single molecule detection (SMD) through associating the target analyte with spatially distinct bright spots observed by fluorescence microscopy. The unspecific interaction of the target analyte and other immunoassay constituents with the sensor surface is of particular interest in this work, as it ultimately limits the performance of this assay. It is minimized by the design of the respective biointerface and thiol self-assembled monolayer with oligoethylene (OEG) head groups, and a poly[oligo(ethylene glycol) methacrylate] (pHOEGMA) antifouling polymer brush was used for the immobilization of the capture antibody (cAb) on the sensor surface. The assay relying on fluorescent postlabeling of long single-stranded DNA that are grafted from the detection antibody (dAb) by RCA was established with the help of combined surface plasmon resonance and surface plasmon-enhanced fluorescence monitoring of reaction kinetics. These techniques were employed for in situ measurements of conjugating of cAb to the sensor surface, tagging of short single-stranded DNA to dAb, affinity capture of the target analyte from the analyzed liquid sample, and the fluorescence readout of the RCA product. Through mitigation of adsorption of nontarget molecules on the sensor surface by tailoring of the antifouling biointerface, optimizing conjugation chemistry, and by implementing weak Coulombic repelling between dAb and the sensor surface, the limit of detection (LOD) of the assay was substantially improved. For the chosen interleukin–6 biomarker, SMD assay with LOD at a concentration of 4.3 fM was achieved for model (spiked) samples, and validation of the ability of detection of standard human serum samples is demonstrated.

## Introduction

The analysis of minute amounts of chemical and biological species serving as biomarkers has become of utmost importance in numerous fields, particularly in the context of early diagnosis, prognosis, and relapse of cancer diseases.^1,2^ The need of novel bioanalytical tools with improved analytical performance characteristics has been addressed by intense research in novel nanomaterials and readout techniques, paving the way toward ultrasensitive biosensors and bioassays.^3,4^ Through rapid advancements in the development of various types of physiochemical transducers and output signal amplification,^5^ detection at the single molecule level has become possible. A generic route to push sensitivity to this ultimate level relies on compartmenting of analyzed sample volume to large series of miniature reactors, in which an enzymatic reaction can generate a measurable signal in the presence of the target analyte.^6,7^ Digital polymerase chain reaction (dPCR, developed for the analysis of nucleic acids) or digital enzyme linked immunosorbent assay (dELISA, introduced for detection of proteins) represent examples where the enzymatically generated output signal allows us to distinguish the presence of individual target molecules against background, given the compartment volume is sufficiently small. By these means, a digital format of the assay readout is established, where counting of individual target molecules provides an accuracy that cannot be reached when the sensor response is averaged over the ensemble of target molecules.

To allow for more efficient multiplexing and open doors for the miniaturization of a digital assay readout, one can exploit an affinity mechanism to capture individual target molecules onto a solid surface from the analyzed liquid sample.^8^ In order to reach sufficient signal-to-noise ratio to distinguish the presence of individual target molecules on the surface, several strategies have been developed, including optical readout in conjunction with amplification reactions or by tightly confining an optically probed volume below the diffraction limit. Among others, there have been reports on the confinement of probing electromagnetic field by resonant coupling to localized surface plasmons supported by metallic nanostructures,^9^ the utilization of low background fluorescence microscopy based on labeling with upconversion nanoparticles,^10,11^ or through associating of sufficient amount of fluorophore emitters at spots where affinity capture of target molecule occurs by rolling circle amplification (RCA).^8^

In general, the performance of all surface-based bioanalytical techniques is ultimately limited by the ability to mitigate unspecific interaction of assay and sample constituents with the sensor surface, leading to a false positive response. In particular, this problem is crucial for the implementation of newly emerging single molecule [single molecule detection (SMD)] techniques for reliable detection of target species present in real samples, including complex biological fluids. Grafting of hydrophilic moieties (typically oligoethylene glycol, or OEG) by using self-assembled monolayers (SAM) is the most commonly used approach to prevent unspecific sorption. While they can achieve significant reduction of nonspecific adsorption of individual proteins, they still suffer from fouling from complex biological fluids such as blood plasma. Significantly improved performance is achieved by polymer brushes, composed of densely packed long hydrophilic polymer chains prepared by “grafting from” the surface.^12^ These are typically accomplished by means of surface-initiated polymerization, such as atom transfer radical polymerization (ATRP) or reversible addition–fragmentation chain transfer polymerization. Coatings with stretched polymer chains exhibiting a thickness between 10 and 70 nm are commonly prepared with zwitterionic groups (e.g., sulfobetaine, phosphorylcholine, and carboxybetaine) and/or noncharged moieties (e.g., hydroxy side groups, OEG side chains, and polyoxazolines). The architecture can consist of simple homopolymer brushes, statistical copolymer/terpolymer-brush systems, block copolymers with varying functionality, or even polyethylene glycol-based pentrimer material modified with functional groups, which have been applied for detection in clinically relevant samples.^13−15^

This work concerns biointerface design that minimizes the background signal in a sandwich immuno-RCA assay in order to allow for the readout of individual affinity-captured target molecules. The performances of both thiol SAMs and a “grafted-from” antifouling polymer brush biointerfaces are benchmarked through the detection of interleukin-6 biomarker. For the first time, the utilization of an antifouling polymer brush biointerface in conjunction with RCA allowing the implementation of SMD of protein biomarker circulating in human serum is demonstrated. After affinity capture, the target analyte is reacted with a detection antibody that is tagged with short oligonucleotide chains. These short oligonucleotide tags are prolonged by RCA producing long ssDNA with repeating sequences serving as multiple labeling sites to yield a strong spatially confined fluorescence signal.^16^ When developing the assay, careful consideration of the biointerface and involved Coulombic interaction associated with negatively charged ssDNA tags appears essential to achieve sensitive detection and prevent false positive signal response. This system displays a sufficient level of sensitivity to visualize individual molecules, thus providing a facile route away from conventional ensemble-averaged signal measurements to SMD.

## Experimental Section

### Materials

From ProChimia surfaces, we used OEG-thiols [OEG–OH, HS-(CH_2_)_11_-EG_6_–OH, prod. no. TH 001-m11.n6], and the OEG-biotin [HS-(CH_2_)_11_-EG_6_-Biotin, prod. no. TH 004-m11.n6] was purchased. The following products were obtained from VWR: phosphate-buffered saline (PBS, pH = 7.4, cat. no. E504), nuclease-free water (NFW, cat. no. E476), Tween 20 (cat. no. 437082Q) and 99.9% pure ethanol (cat. no. 1.11727), calcium chloride (CaCl_2_, cat. no. C1016), sucrose (cat. no. S7903), and dibenzocyclooctyne-*N*-hydroxysuccinimidyl ester (DBCO-NHS, cat. 761524), and poly(ethylene glycol) methacrylate (HOEGMA, *M*_n_ = 5 00 g/mol), copper(I) chloride (CuCl, ≥99.995% trace metals basis), copper(II) bromide (CuBr_2_, 99.999% trace metals basis), 2,2′-bipyridyl (99%), 4-(dimethylamino)pyridine (≥99%), *N*,*N*′-disuccinimidyl carbonate (≥95%), and dithiothreitol (DTT, cat. 43,815) were ordered from Sigma-Aldrich. *N*,*N*-dimethylformamide (DMF, 99.8%, extra dry over molecular sieves) was purchased from Lach-Ner, Czech Republic. The ATRP initiator thiol ω-mercaptoundecyl bromoisobutyrate was synthesized according to a previously published procedure.^17^

Bovine serum albumin (BSA, cat. no. B9000S) was obtained from New England Biolabs. Ampligase DNA ligase with buffer (cat. no. A3202K) were obtained from LGC Genomics GmbH. The following products were purchased from Thermo Fisher Scientific: NeutrAvidin (NA, cat. no. 31050), streptavidin (SAv, cat. no. 434301), FastAP Thermosensitive alkaline phosphatase (cat. no. EF0651), Exonuclease I (Exo I, cat. no. EN0581), deoxy nucleoside triphosphates (dNTPs, cat. no. R0192), and phi29 DNA polymerase (φ-29 Pol, cat. no. EP0094), as well as Zeba spin desalting columns (7k MWCO—cat. 89,882 and 40k MWCO—cat. 87,766) and sulfo-SMCC (sulfosuccinimidyl 4-(*N*-maleimidomethyl)cyclohexane-1-carboxylate–cat. 22,322).

The anti-IL6MQ2–13A5 (cat. 14-7069-85), MQ2-39C3 (cat. 13-7068-85), and anti-TNF-alpha mAb11 (cat. 13-7349-85) were also obtained from Thermo Fisher Scientific, while the MQ2-13A5-Alexa647 (cat. 501124) was purchased from BioLegend UK Ltd. The recombinant human IL-6 protein (cat. ab198571) was ordered from Abcam plc. All DNA sequences (summarized in Table 1) were purchased from Integrated DNA Technologies. From Randox (UK) standard serum samples were acquired with human IL-6 concentrations of 15.36, 194.44, and 606.86 pg/mL.

**Table 1:**
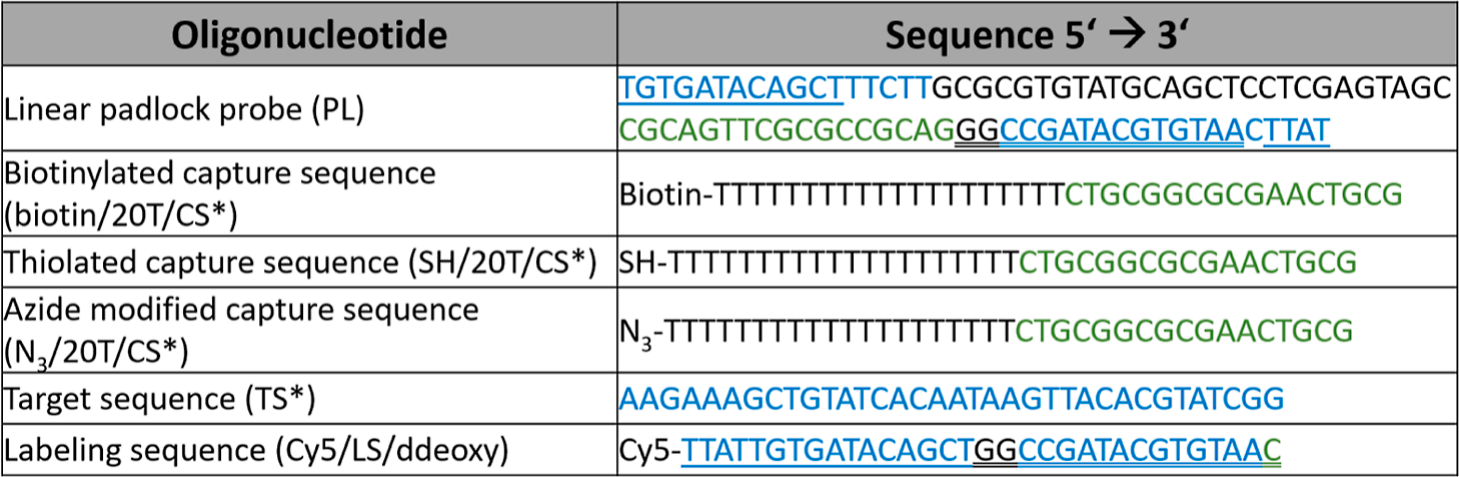
Summary of DNA Sequences Where (*) Indicates Complementary Sequence to Padlock Probe PL; Colors Mark the Specific Parts of Sequences, and Underlined Are the Respective Complementary Parts of Sequences

### Preparation of Biointerfaces on Gold Sensor Chips

Glass substrates (BK7 or LASF9) were thoroughly cleaned in ultrapure water (*R* ≥ 18.2 MΩ/cm^2^), a 1% (v/v) solution of Hellmanex III and ethanol for a period of 15 min of sonication at 60 °C. Afterward, the slides were rinsed with pure ethanol, dried with pressurized air, and placed on a rotary stage in a vacuum thermal evaporator (HHV Ltd., Auto306 Lab Coater, UK), after which 2 nm of chromium and 50 nm of gold were deposited under high vacuum. An ethanolic solution was prepared with 0.2 mM of thiols with a biotin headgroup and 0.8 mM with OEG groups, in which the gold slides were incubated for at least 1 day. Then, the slides with formed mixed thiol SAM were rinsed with pure ethanol, dried with nitrogen, and stored in the dark under an argon atmosphere until use.

For the polymer brush synthesis (schematically shown in Figure S1), the gold-coated substrates were first rinsed with ethanol and deionized water, dried with nitrogen, activated in a UV/O_3_ cleaner for 20 min, immediately immersed in a 1 mM solution of ATRP initiator ω-mercaptoundecyl bromoisobutyrate, and incubated overnight in the dark. After removal from the thiol solution, the substrates were rinsed with ethanol and water, dried with nitrogen, and placed in sealed reactors that were purged with argon. In a separate flask, HOEGMA (15 g, 30 mmol), CuBr_2_ (12.2 mg, 0.054 mmol), 2,2′-bipyridyl (217.5 mg, 1.39 mmol), and water (15 mL) were deoxygenated by bubbling of argon under stirring for 1 h, after which CuCl (55.5 mg, 0.561 mmol) was added under argon, and the mixture was stirred until dissolution. The polymerization solution was then transferred under argon to the substrate-containing reactors. After 25 min of reaction, the polymerization was stopped by the addition of nondegassed water, where substrates were rinsed with ethanol and deionized water and dried with nitrogen.

Immobilization of SAv was performed according to a published procedure.^18^ The pHOEGMA-coated samples were first sealed in reactors and purged with argon. 4-(dimethylamino)pyridine (72 mg, 0.6 mmol) and *N*,*N*′-disuccinimidyl carbonate (153 mg, 0.3 mmol) were dissolved in anhydrous DMF and the solution was added under argon protection to the reactors, which were kept in the dark overnight. The samples were subsequently removed from the reactors, rinsed with DMF (6 mL) and deionized water, dried with nitrogen, covered with a solution of SAv (100 μg/mL) in PBS and kept for 1 day at 4 °C in a humidity-controlled chamber. The pHOEGMA-SAv samples were then rinsed with copious amounts of PBS and stored in PBS in the fridge.

### Circular Padlock

The linear PL strand was circularized at the 5′ and 3′ ends in an enzymatic ex situ ligation reaction with the sequence (TS*) binding to 17 or 18 bases on each end followed by exonuclease treatment to remove all nonligated DNA.

The ligation of the linear padlock probe was performed by reacting 75 units of DNA ligase, 40 nM of the target sequence TS* and the ligation buffer (20 mM Tris–HCl, 25 mM KCl, 10 mM MgCl_2_, 0.5 mM NAD, and 0.01% Triton X-100) in NFW-BSA (0.2 mg/mL) in a total volume of 250 μL at 50 °C for 1 h. Each step of the procedure was performed on a thermomixer at 700 rpm, and the solution was stored on ice in between reaction steps. The reaction was terminated by raising the temperature to 85 °C for 15 min.

50 units of exonuclease I and 5 units of alkaline phosphatase were added in the respective buffer (67 mM glycine-KOH, 6.7 mM MgCl_2_, 1 mM DTT) to the ligation mixture with the circularized padlock probe in a total volume of 500 μL. The reaction was conducted at 37 °C for 15 min, and it was terminated by raising the temperature to 85 °C for 15 min.

### Coupling of ssDNA Tag to Detect Antibody dAb

For the experiments on the carboxy-SAM, the clone MQ2-39C3 (0.5 μg/mL in PBS) was purchased as biotinylated or unconjugated for the conjugation with the DBCO-NHS ester (Figure S2b), while for the biotin-SAM, the clone MQ2–13A5 (0.5 μg/mL in PBS) was conjugated with sulfo-SMCC ester (Figure S2c). The ester was dissolved in DMSO at a molar concentration of 10 mM, added in 100 or 80 molar excess to the anti-IL6 and incubated on a Hula-mixer for 1 h at room temperature following the manufacture’s procedure. Excess molecules were removed with spin desalting columns (40k MWCO) after the reaction. The thiolated primer sequence was prepared by dissolving in 1× TE buffer with DTT (10 mM) for reduction of the disulfide groups according to the recommendations of the manufacturer. The solution was passed through a spin desalting column (7000 MWCO) for the removal of DTT immediately before the addition of the DNA to the SMCC-antibody solution in a molar excess of 25. The reaction was incubated on a Hula-mixer for 3 h at room temperature and stopped by the removal of excess molecules with a spin desalting column (40k MWCO). The anti-IL6/DNA complex was stored at −20 °C until use.

### Immuno-RCA Assay

Modified gold sensor chips carrying mixed thiol-SAM of OEG and biotin or carboxyl headgroups with a molar ratio of 1:5 were used for each experiment. The working buffer was PBS with 0.01% Tween20 (PBST). The biotin-SAM was first contacted for 20 min with a solution containing NA (1.87 μM in PBST) to which the biotinylated capture antibody cAb MQ2-39C3-biotin (25 μg/mL in PBST) binds upon a subsequent 20 min reaction. On the activation step of EDC/NHS (200 mM/50 mM) in NFW for 10 min was used prior to the reaction with cAb. Then, sodium acetate buffer (ACT) was flowed for 1 min, and the capture antibody MQ2-13A5 (50 μg/mL) in ACT buffer with pH 5.55 was immobilized by a 20 min long amine coupling reaction. Afterward, ethanolamine (1 M in NFW) was used for the deactivation of the active groups for 20 min.

The interleukin-6 analyte diluted in PBST [for surface plasmon resonance (SPR)/plasmon-enhanced fluorescence (PEF) and for SMD with 0.2 mg/mL BSA] with varied concentrations was flowed over the surface for 10 min. Standard serum samples were diluted 10-times with the working buffer. Subsequently, in the case of the biotin-SAM, the premodified detection antibody MQ2-13A5/CS- was flowed for 20 min (1 μg/mL in different buffers) over the surface, while MQ2-39C3/DBCO (1 μg/mL in PBST for 20 min) or MQ2-39C3/biotin and streptavidin-A647 (1 μg/mL in PBST for 20 min) was used for the carboxy-SAM experiments with a subsequent step of flowing the capture sequence modified with an azide-group or biotin-tag (40 nM in PBST) for 25 min.

The hybridization of padlock probe PL was conducted for 40 min in ligation buffer. Then, the RCA was conducted by a mixture of φ29-polymerase (100 units) and dNTPs (100 μM) in the respective buffer (33 mM Tris-acetate, 10 mM Mg-acetate, 66 mM K-acetate, 0.1% Tween 20, and 1 mM DTT). After 15 to 60 min, PBST was rinsed to terminate the amplification reaction. The labeling oligonucleotides with Cy5-tags (10 mM in PBST) were then introduced for 15 min. Between each immobilization step, the working buffer was rinsed for 5 min.

### Optical SPR/PEF Setup

**Figure 1:**
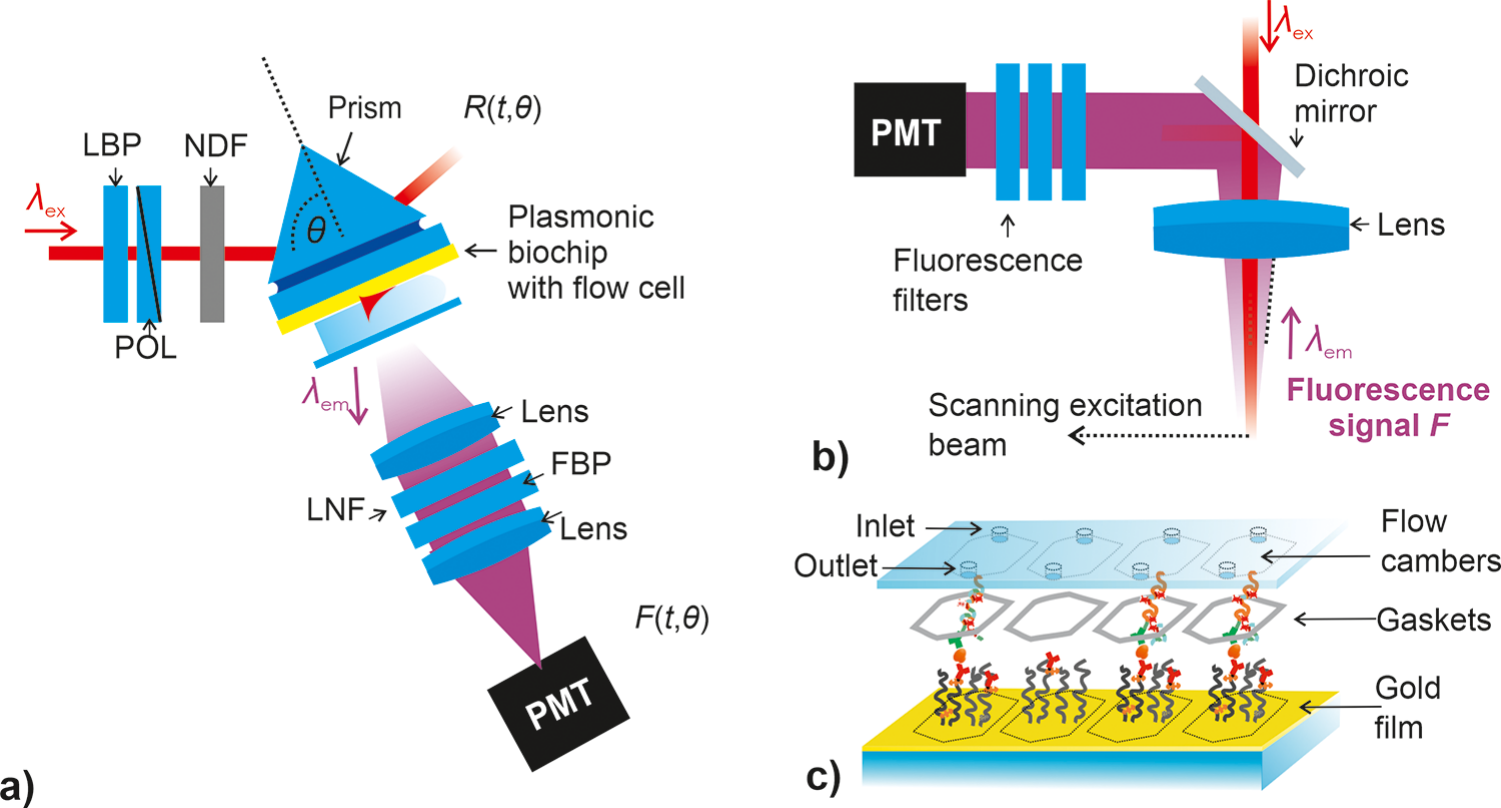
Schematics of (a) optical system used for the combined SPR/PEF measurements and of (b) epi-illumination fluorescence scanning and (c) a sensor chip with four flow chambers.

For the SPR/PEF measurements, a home-built setup based on the Kretschmann configuration of attenuated total reflection method was used (see Figure 1a). The sensor chip composed of a glass substrate with a thin gold film was optically matched with a prism made from LASFN9 glass by using an immersion oil with refractive index *n* = 1.7000 (Cargille Laboratories, USA). Excitation light beam with transverse magnetic polarization that was emitted from a HeNe laser (λ_ex_ = 632.8 nm) was spectrally cleaned with band-pass filter (LBP, from Thorlabs, UK) and its intensity was adjusted with a set of neutral density filters (NDF, from Thorlabs, UK). The beam was made impinging onto the gold-coated sensor surface under an angle of incident θ that was controlled with a motorized rotation stage (from Huber GmbH). The reflected beam intensity *R* (in %) was measured by a photodiode that was connected to a lock-in amplifier. A flow cell was clamped against the sensor surface to create a reaction chamber with a total volume of 10 μL defined by using a thin PDMS gasket sealed by a glass substrate with prepared inlet and outlet holes connected to Tygon tubings (inner diameter of 0.64 mm). Peristaltic pumping was employed to transport the sample solutions in a closed loop at a constant flow rate of 20 μL/min.

A photomultiplier tube (Hamamatsu, H6240-01) and counter (Agilent, 53131A) served for the detection of fluorescence light intensity emitted from the sensor surface (at λ_em_ = 670 nm) through the transparent flowcell upon the probing with resonantly excited surface plasmons (at λ_ex_ = 632.8 nm). The fluorescence beam was collimated by a lens (Thorlabs, focal length *f* = 50 mm, numerical aperture of NA = 0.2, LB1471), spectrally cleaned by a laser notch filter (LNF, Melles Griot, XNF-632.8–25.0 M CVI), and two bandpass filters (FBPF, Thorlabs, FB670-10 and Andover Corporation Optical Filter, 670FS10-25) prior to its focusing at the photomultiplier tube entrance. The output fluorescence signal *F* (measured in counts per seconds—cps) and reflectivity intensity *R* were recorded in time by using Wasplas software (developed at Max Planck Institute for Polymer Research in Mainz, Germany).

### Evaluation of SPR/PEF Data

Angular reflectivity scans *R*(θ) were recorded for gold coated slides with dry surface modification and when contacted with a buffer before the start of each kinetics measurement. The SPR response *R*(*t*) was measured at the angle of incidence θ set fixed at the linearly decreasing slope of the SPR dip. At the end of each experiment the SPR response *R*(*t*) was recorded for subsequent flow of sucrose dissolved in PBST at 1, 2, and 4% (w/w) concentration with corresponding bulk refractive indices of *n*_b_ = 1.3344, 1.3359, and 1.3388 in order to convert the signal to refractive index unit. The noise σ of the measurements was deduced from the angular fluorescence scans *F*(θ) at the dip of the surface plasmon taken after the labeling procedure. By establishing a calibration curve, the limit of detection (LOD) could be determined with the fluorescence response Δ*F*_b_ from the control experiment plus three times the noise σ.

### Multichannel Microfluidics and Fluorescence Read-Out

After the immuno-RCA assay, the surface was contacted with glutaraldehyde in citric acid buffer (100 mM, pH = 7) for 30 min serving as fixative. Subsequently, the sensor chip was imaged in PBST with the Olympus FV1000 confocal fluorescence microscope (see Figure 1b) with a magnification of 4×, 10×, and 40× for SMD, which were then loaded into the ImageJ software for defining the threshold and counting of particles. The threshold was determined by plotting the histograms and determining the strongest changes with the analyte concentration. The LOD was calculated with the calibration curve and the number of spots *N*(0) + 3 × σ deduced from the images with 40× magnification of different areas in the channel.

SMD experiments were implemented by using a multichannel flow cell that was designed to accommodate the delivery of solution over the sensing surface in a four-channel parallel arrangement (see Figure 1c). The flow cell housing was printed using stereolithography (Prusa Sl1s) using a commercially available resin (Prusa green transparent) and default printer settings. After printing, the part was rinsed thoroughly with isopropyl alcohol (with forced flow through each microchannel), dried with nitrogen, and cured with UV. The flow cell had interface ports to accommodate a short section of Tygon tubing (ID 0.64 mm, sealed with resin via UV curing), where internal microchannels of 0.5 mm diameter led to a flat sealing face situated directly opposite the sensing surface. Sealing was accomplished via a single side self-adhesive polished vinyl gasket (thickness 0.09 mm) that was fixed directly to the flow cell. The system was sealed to the sensing surface by using a custom-built clamping system, where flow was achieved by a peristaltic pump.

## Results and Discussion

**Figure 2:**
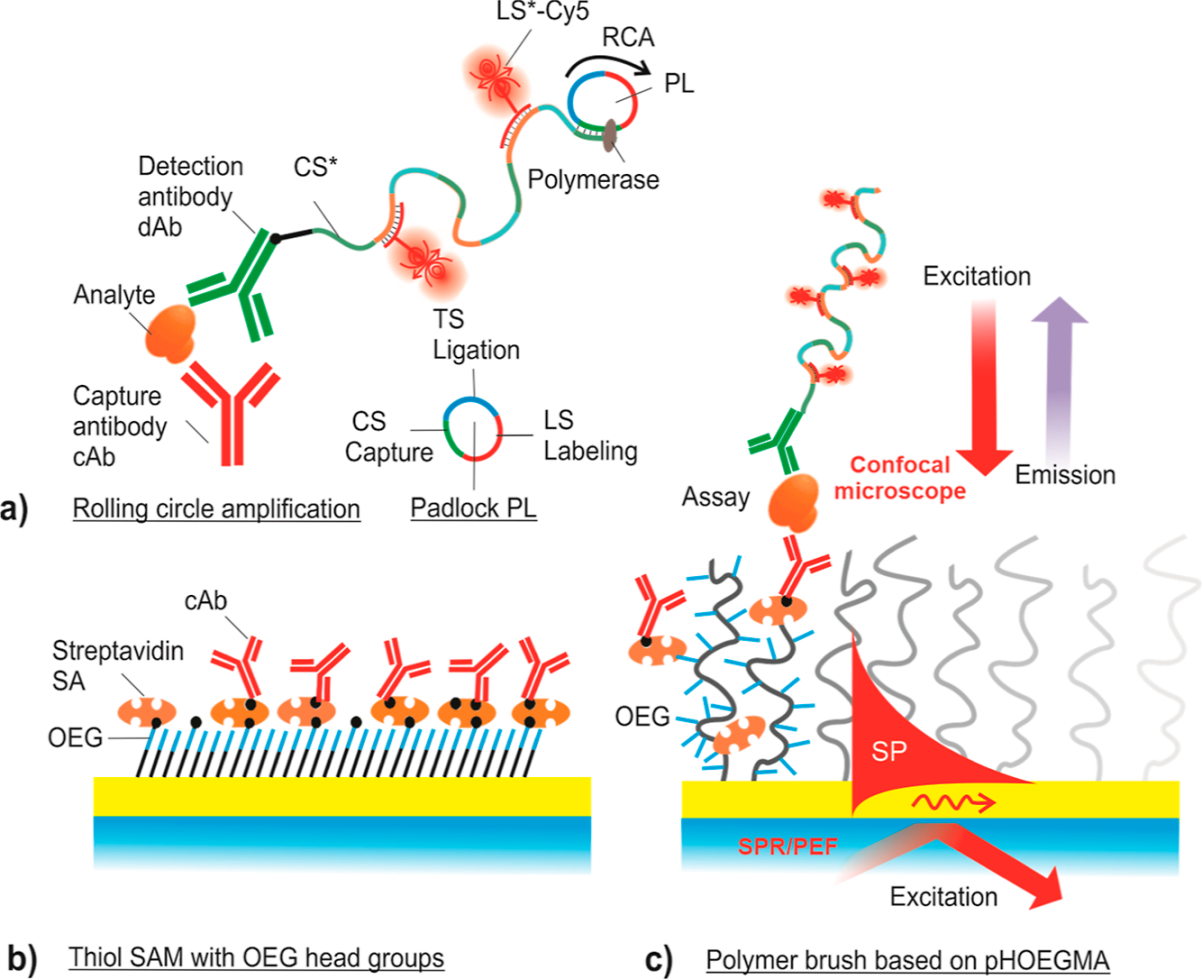
(a) Schematics of sandwich immunoassay with RCA initiated at a biointerface carrying capture antibody cAb anchored on (b) thiol SAM with OEG headgroups and (c) on pHOEGMA polymer brush.

There were carried out experiments on the implementation of sandwich immuno-RCA on a solid sensor surface for the detection of protein analytes at the single molecule level. As illustrated in Figure 2, the biointerface design is based on either biofunctional thiol SAMs or polymer brushes to anchor the capture antibody (cAb) to the sensor surface, used in conjunction with the detection antibody (dAb) tagged with a short ssDNA sequence. Building up on our previous works,^16,19^ this tag allowed for RCA growths of >1 μm long ssDNA chains that can be postlabeled by hybridization with a large amount of short ssDNA strands carrying Cy5 fluorophores (>10^2^ emitters/RCA-generated chain). The successful RCA reaction and the ability to generate long (>10^4^ bp) DNA chains was also tested in solution and visualized via agarose gel-electrophoresis, as shown in Figure S3 for RCA reaction times of *t* = 10 min, 20 min, 30 min, and 1 h.

The fluorescence readout of the assay was first established by using combined SPR and PEF measurements, with the optical signal averaged over a ∼mm^2^ area carrying an ensemble of target molecules. Afterward, fluorescence imaging of the sensor surface was utilized for the detection of individual target molecules. A confocal fluorescence microscope was used and the SMD analysis of acquired images was performed based on counting bright spots associated with the specific capture of the target analyte. This approach was tested for spiked buffer as well as for human serum samples with the whole assay being implemented in a multichannel microfluidic device for processing of multiple samples in parallel.

### Immuno-RCA Procedure

As a model analyte, interleukin-6 (IL-6) was used in the assay. It is an inflammatory biomarker that also plays an important role in the innate and adaptive immune system, and its expression is associated with various cancers. It should be noted that the reported method is not restricted to this analyte and it can be implemented for a wide range of other protein analytes with established sandwich immunoassays, such as cytokines, vascular endothelial growth factor, or tumor necrosis factor alpha (TNF-alpha).

After the capture of IL-6 on the sensor surface bearing capture antibody cAb, detection antibody dAb was reacted with the analyte to form a sandwich. dAb was tagged with a short ssDNA sequence CS* (see Table 1) that is complementary to a segment CS of the circular padlock probe PL. After forming the sandwich, CS* attached to dAb was hybridized with PL and the RCA process was initiated by flowing a solution containing the φ29-polymerase docking at the 3′ end of CS*. Gradual incorporation of nucleotides (dNTPs) leads to the prolongation of CS* ssDNA chains with the repeating reverse-complementary sequences of the PL (see Figure 2a). The construct is then used to accommodate multiple labeling sequences LS-Cy5 (see Table.1). They are designed to bind to two distinct locations of the long RCA-generated ssDNA strands to compact them and thus confine the generated bright fluorescence spots.^19^ For the purpose of comparison, the assay was deployed on a gold surface functionalized with either a thiol-based SAM carrying OEG headgroups (see Figure 2b) or with a pHOEGMA polymer brush (see Figure 2c). In order to couple cAb with the surface, we employed both the Avidin–biotin interaction (biotin-SAM surface and pHOEGMA brushes) or amine coupling (carboxy-SAM). In addition, several conjugating chemistries for tagging CS* to dAb were tested based on maleimide, click, and avidin–biotin reactions in order to reach maximum specific sensor signal against the background.

### Monitoring of Immuno-RCA by SPR/PEF Biosensor

The attachment of biomolecules to the surface and the RCA growth of long ssDNA chains was monitored in real-time by a combined SPR and PEF setup that enables the parallel measurement of both surface mass density changes and fluorescence signal associated with the incorporation of fluorophore-tagged molecules to the surface. Both readout modalities are sensitive to changes occurring within a very close proximity to the surface, which is probed by confined electromagnetic field of resonantly excited surface plasmon waves (to a distance of ∼100 nm).

**Figure 3:**
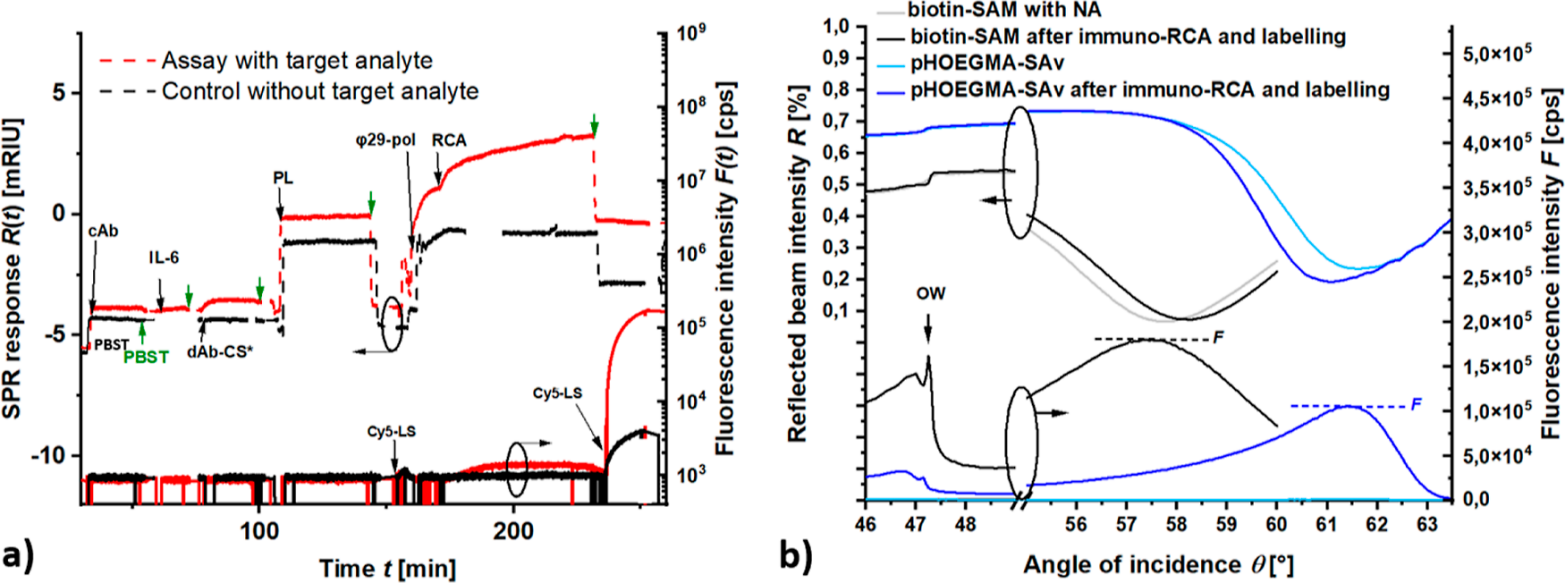
(a) Combined SPR/PEF readout of the sandwich immunoassay RCA kinetics for a sample with target analyte (IL-6 concentration *c* = 47.6 nM) and blank sample (IL-6 concentration *c* = 0). (b) Comparison of angular reflectivity *R*(θ) and fluorescence *F*(θ) scans measured for the IL-6 immuno-RCA assay on a thiol SAM and pHOEGMA brush interfaces with cAb-biotin and dAb-Cy5 prepared by maleimide conjugation chemistry. OW marks the angle where the resonant excitation of the dielectric optical waveguide mode occurs.

Figure 3a shows an example of the SPR/PEF sensorgram for the immobilization of cAb, affinity capture of IL-6 and dAb-CS*, as well as the RCA reaction and postlabeling step. In this experiment, a mixed thiol SAM carrying biotin and OEG headgroups was prepared on the gold sensor surface. The NeutrAvidin—NA—immobilization increased the SPR sensor response by Δ*R*_a_ = 1.27 mRIU, which corresponds to the surface mass density of 0.59 ng/mm^2^ and is close to a fully packed monolayer.^20^ As Figure 3a shows, the subsequent coupling of biotin-cAb caused the SPR signal change of 1.53 mRIU that suggests that about 50% of NA on the surface reacted with biotin-cAb (taking into account molecular weights of NA of 67 kDa and IgG of 150 kDa). Afterward, buffer solution spiked with IL-6 analyte at a concentration of *c* = 47.6 nM was reacted with the surface, leading to a shift of Δ*R*_a_ = 0.08 mRIU. This value corresponds to a near saturation of the specific binding sites, as about 40% cAb reacted with the target analyte (IL-6 molecular weight of 21 kDa). A sandwich was then formed by incubating the surface with dAb-CS* (conjugated with maleimide-based chemistry; see Figure S2c) showing a SPR signal change of Δ*R* = 0.31 mRIU, which indicates the occupation of about 50% of the captured IL-6. The padlock PL was then loaded to the sensor with a molar concentration of *c* = 40 nM in the ligation and exonuclease buffer with a different bulk refractive index, leading to an abrupt jump in the sensor output. Afterward, the surface was contacted with the RCA solution containing only φ29-Pol in the respective buffer. After 10 min, the RCA reaction was started by adding dNTPs and the process was terminated after 60 min by flushing with working buffer, showing an increase in *R*(*t*) of Δ*R* = 3.6 mRIU. This response is similar to that observed in our previous study on dense ssDNA brushes^16^ indicating an efficient capture of target analyte that herein initiates the growth of ssDNA chains. Finally, the short Cy5-LS probes were flowed over the surface, the hybridization with RCA generated ssDNA chains is accompanied by a strong fluorescence increase of Δ*F* = 1.73 × 10^5^ cps.

In a control experiment, the same procedure was performed albeit without incubating the sensor surface with IL-6. The acquired data presented in Figure 3a show a nonmeasurable SPR response due to the binding of dAb-CS* and a 65-fold lower fluorescence signal of Δ*F* = 2.66 × 10^3^ cps after the RCA reaction and post labeling step. Interestingly, a substantial SPR signal increase of Δ*R*′ = 1.84 mRIU was observed after the RCA in the control experiment that could be attributed to unspecific sorption of the RCA reaction constituents. However, in the fluorescence experiment, they impose a negligible effect as they do not lead to the prolongation of the CS* chains and the labeling Cy5-LS probes do not react with the surface (see the control data in the fluorescence channel measured prior to the RCA as presented in the bottom part of Figure 3a).

It is worth of noting that the RCA-generated ssDNA chains exhibit highly extended polyelectrolyte brush architecture for sandwich immunoassay with high nM concentration of target IL-6 analyte (similar to our previous report when CS* sequences were directly immobilized on the thiol SAM surface^16^). The presence of optical waveguide mode (OW) in the angular fluorescence *F*(θ) and reflectivity *R*(θ) scans (see narrow resonant features occurs close to the critical angle θ_c_Figure 3b) confirms that the chains stretch to ∼μm distances, as the dielectric adlayer formed by the RCA can act as an optical waveguide. Importantly, such characteristics were sustained when the IL-6 immuno-RCA assay was implemented on the pHOEGMA—based biointerface carrying SAv. Figure 3b compares the angular scans measured before and after the immuno-RCA assay on the thiol-SAM and pHOEGMA biointerfaces (the respective SPR/PEF sensorgram is shown in Figure S4a). The pHOEGMA biointerface exhibits a thickness of *d* = 32 nm in the dry state, which is expected to increase by between 1.5 and 2 times when swollen in aqueous buffer.^12,22^ The immobilized SAv is assumed to be present at the upper interface between the pHOEGMA and aqueous buffer as well as inside the layer with a gradient toward the inner gold interface. The corresponding peak fluorescence signal *F*(θ) measured after the immuno-RCA and following reaction with Cy5-LS was Δ*F* = 1.05 × 10^5^ cps, which is comparable to the value measured on thiol SAM and it proves that RCA can tolerate the presence of densely packed pHOEGMA polymer chains. The occurrence of SPR at angle shifted by about 3.5 deg is ascribed to the increased refractive index and thickness of the pHOEGMA biointerface compared to the thiol SAM.

### Calibration Curve for Ensemble-Averaged Response

**Figure 4:**
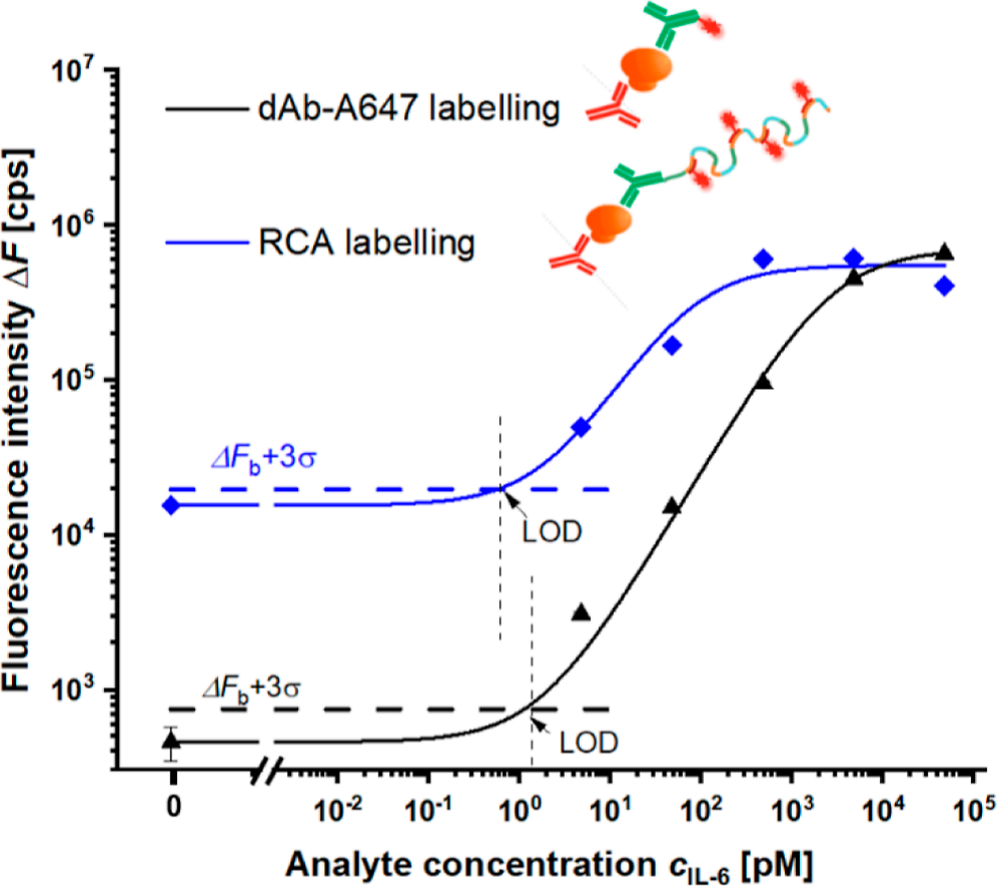
Comparison of calibration curves obtained for the PEF readout of IL-6 sandwich immunoassay on a thiol SAM (i) without RCA and labeling of dAb-Alexa647 and (ii) with RCA and labeling by LS-Cy5.

Figure 4 shows calibration curves measured for varied concentrations of IL-6 and PEF readout of the immuno-RCA assay, when the output fluorescence signal is averaged over the surface area of several mm^2^. It compares the fluorescence intensity Δ*F* measured for the assay format with the directly labeled dAb and for the post labeling of immuno-RCA product when dAb conjugated with CS* is used. For the direct labeling, the experiments were conducted on the biotin-SAM with biotinylated cAb anchored via NA and the dAb conjugated with Alexa647-fluorophores. The immuno-RCA was performed on a surface with carboxy-SAM carrying cAb anchored by amine coupling, where the dAb-biotin was linked with the biotin-CS* via SAv, and the postlabeling was utilized by Cy5-LS. Both assays were tested for samples with IL-6 dissolved at a concentration between *c* = 4.8 pM and 48 nM and Δ*F* was obtained close to the angle where the SPR dip occurs.

The acquired calibration curves reveal that the fluorescence signal Δ*F* increases with IL-6 concentration *c* and it saturates at similar intensity close to 5 × 10^5^ cps for both assays. Interestingly, the response of immuno-RCA assay reaches saturation at lower IL-6 concentrations (*c* ∼ 1 nM) when compared to the assay with direct labeling (*c* ∼ 10 nM). This can be explained by the effect of the ssDNA brush architecture taken by long RCA-generated chains that repel each other at sufficiently high grafting density. It occurs at high analyte concentrations *c* and the RCA process leads to the generation of ssDNA chains stretched to a distance >1 μm from the sensor surface, pushing their substantial portion outside the evanescent probing field of surface plasmons, which does not contribute to the enhancement of fluorescence signal Δ*F*. The RCA chains are expected to take a random coil conformation for lower IL-6 concentrations, where they then fold within the surface plasmon evanescent field, and the enhancement provided by the immuno-RCA assay format is more pronounced when compared to the direct labeling of dAb. Accordingly, Figure 4 shows that the enhancement factors of 11.1 and 16.0 were obtained for low IL-6 concentration of *c* = 47.6 and 4.76 pM, respectively. Unfortunately, this enhancement translated to only minor 2.1-fold improvement of the LOD (yielding of 0.5 pM) of immuno-RCA with respect to the assay with direct dAb labeling. This observation is contrary to previous work, where RCA was used for the detection of nucleic acid–based analytes providing 100-times better LOD.^16^ As can be seen in Figure 4, the major problem is that the immuno-RCA is accompanied by > 20 times increased background fluorescence signal, which can mainly be attributed to the unspecific sorption of dAb-CS* conjugate to the surface.

### Minimizing Background Signal

In order to suppress the RCA-enhanced background signal that masks the specific response and thus severely impairs the performance of the assay, we first tested the impact of the conjugation chemistry used for both the anchoring of cAb to the sensor surface and the attachment of CS* to dAb. Similar to results shown in Figure 3a, we quantified the performance of the tested combinations by using the ratio of the fluorescence signal increase measured for specific (Δ*F*_a_, IL-6 concentration of *c* = 47.6 nM) and control assay (Δ*F*_b_, background *c* = 0). On the carboxy-SAM, cAb was immobilized by amine coupling, and the biotinylated dAb was conjugated with biotin-CS* via SAv (configuration 1) or dAb was modified with DBCO-groups for clicking to the CS* carrying an azide end group (configuration 2). The biotin-SAM was utilized for the immobilization of cAb bearing biotin groups via a NA linker, and the dAb was modified by maleimide groups to which CS* were covalently bound via their thiolated end group (configuration 3). The ratio of the fluorescence signal Δ*F*_a_/Δ*F*_b_ was measured for dAb dissolved in PBST by using the PEF readout.

**Figure 5:**
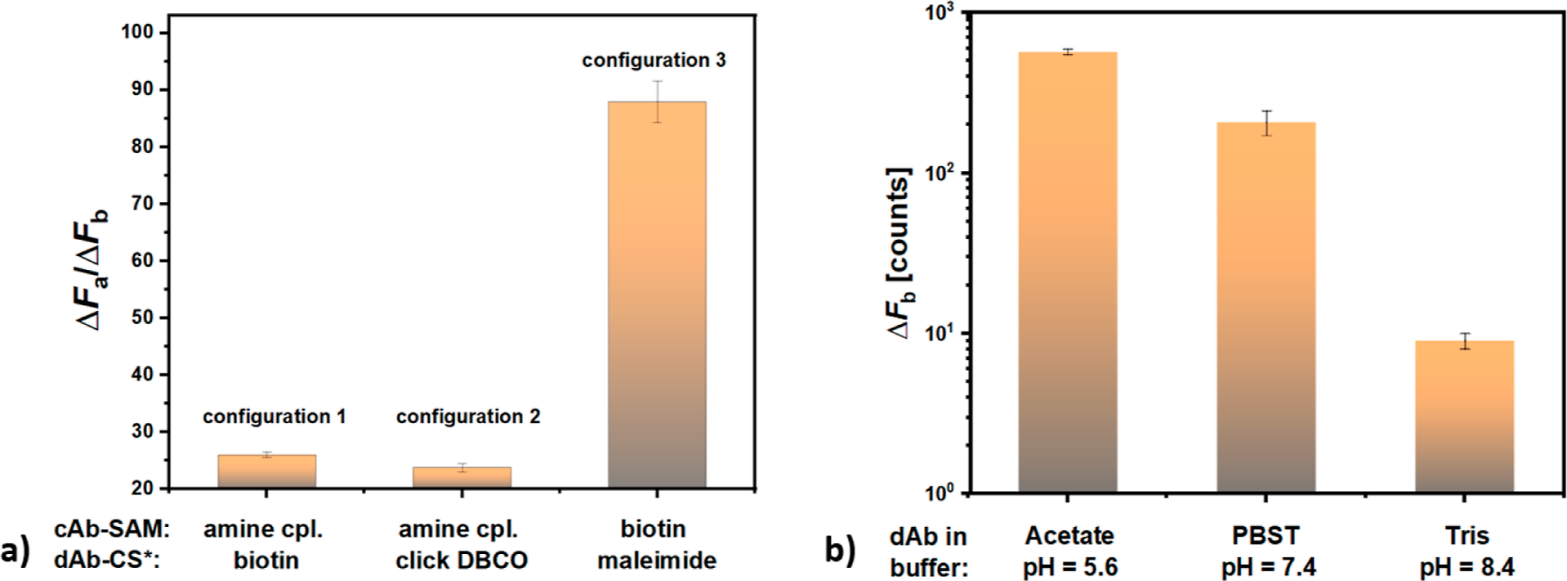
(a) Comparison of fluorescence intensity ratio for sandwich immunoassay with RCA measured for a sample with target IL-6 concentration of 47.6 nM and for blank sample Δ*F*_a_/Δ*F*_b_ depending on the conjugation chemistry used for anchoring cAb to the gold surface and tagging of CS* to dAb (data were measured with PEF for dAb dissolved in PBST). (b) Fluorescence intensity measured for a blank sample Δ*F*_b_ depending on the buffer pH with cAb anchored by amine coupling and dAb tagged with CS* by maleimide chemistry.

Results summarized in Figure 5a show that configuration 1 demonstrated a low Δ*F*_a_/Δ*F*_b_ ratio of 26, which was ascribed to a strong unspecific binding of dAb-biotin or SAv to the biointerface. In order to mitigate the possible effect of unspecific sorption of SAv and reduce the number of steps in the assay, an alternative configuration 2 with the dAb modified by CS* via the click chemistry was utilized. Interestingly, this yielded a similar ratio of Δ*F*_a_/Δ*F*_b_ = 23.8, suggesting that the interaction with dAb with the cAb-modified carboxy-SAM causes the problem rather than using the SAv linker. The use of configuration 3, consisting of a biotin SAM and dAb-CS* prepared by maleimide chemistry, improved the ratio to Δ*F*_a_/Δ*F*_b_ = 88 and therefore was used for all further experiments.

In order to further decrease the acquired background signal *F*_b_, we investigated the effect of the buffer conditions used for the reaction of the surface with dAb. The control assay was tested for the biotin-SAM with the dAb-CS* complex prepared by the maleimide coupling (for configuration 3) and dAb conjugate was dissolved at the same concentration in buffers with variable pH: pH = 5.6 (sodium acetate with 25 mM and sodium chloride with 50 mM); pH = 7.4 (PBST) and pH = 8.4 (Tris–HCl with 10 mM and sodium chloride with 50 mM). The obtained results presented in Figure 5b reveal that the pH-dependent net charge of dAb and of the biointerface bearing cAb plays an important role in the strength of the background signal *F*_b_. At pH = 8.4, the background fluorescence signal was 22 times lower than that measured for PBST (pH = 7.4). Contrary to this, when decreasing pH to 5.6, the background signal increased by a factor 2.9 with respect to PBST with pH = 7.4. A net charge of proteins depends on its isoelectric point^23^ and the herein used IgG antibodies have been reported to exhibit pI in the range from pH = 6.1 to 9.1.^24^ Since dAb was coupled to a DNA strand with a negative phosphate backbone, the pI of dAb-CS* can be expected in the lower range with stronger negative charge at the higher pH. The minimized background response at an increase pH can be explained by the effect of weak Coulombic repelling between the dAb and the surface. Since using this condition allowed us to push the Δ*F*_a_/Δ*F*_b_ > 10^3^ for the biointerface with cAb-biotin, dAb was further reacted with the surface when dissolved in Tris pH = 9.1.

It should be noted that in addition to the thiol SAM architectures, we also tested poly[(*N*-(2-hydroxypropyl)-methacrylamide)-*co*-(carboxybetaine methacrylamide)] (HPMA-*co*-CBMA) brushes for minimizing background signal *F*_b_. This polymer coating takes advantage of zwitterionic groups that tightly bind water molecules, where such surfaces have been reported to exhibit ultralow fouling properties, even after the postmodification with protein ligands by using amine coupling.^25^ However, the obtained results (see Figure S5a for testing various deactivation agents and Figure S5b for dAb-CS* complex dissolved in different buffer solutions) did not reveal such functionality, and sufficient repelling from unspecific interaction of dAb-CS* conjugate was not achieved when IL-6 immuno-RCA assay was applied. It was ascribed to unbalanced charge density occurring after the cAb was attached to the surface via the carboxy-betaine groups with the active ester chemistry (leading to net positive charge and possible Coulombic interaction with negatively charged dAb-CS*). Therefore, to minimize the background signal *F*_b_, the coupling chemistry based on the biotin tags attached to cAb was implemented with pHOEGMA brushes that carry uncharged OEG groups. pHOEGMA were postmodified by SAv and together dAb-CS* prepared by maleimide chemistry; this interface was adapted for the best performing configuration 3.

### Digital Readout of Immuno-RCA Assay

The SMD experiments were performed with interfaces that allowed for minimizing the background signal *F*_b_. For the initial analysis of samples where PBST was spiked with target IL-6 analyte at concentrations of *c* = 0 48, 480, 4.8, 48, and 143 pM, a biotin-SAM interface was used. The assay was implemented by using dAb-CS* diluted in Tris–HCl (pH = 8.4), PL with a molar concentration of *c* = 40 nM, and RCA time of 60 min. After the labeling of the RCA product with Cy5-LS, glutaraldehyde dissolved in CB buffer was flowed over the surface functioning as a fixative. The experiments were carried out by using a four-channel microfluidic device to perform multiple experiments on a chip with one channel dedicated as a control. The overall assay including the flow of analyzed sample, reaction with dAb, RCA, and postlabeling step required 145 min.

**Figure 6:**
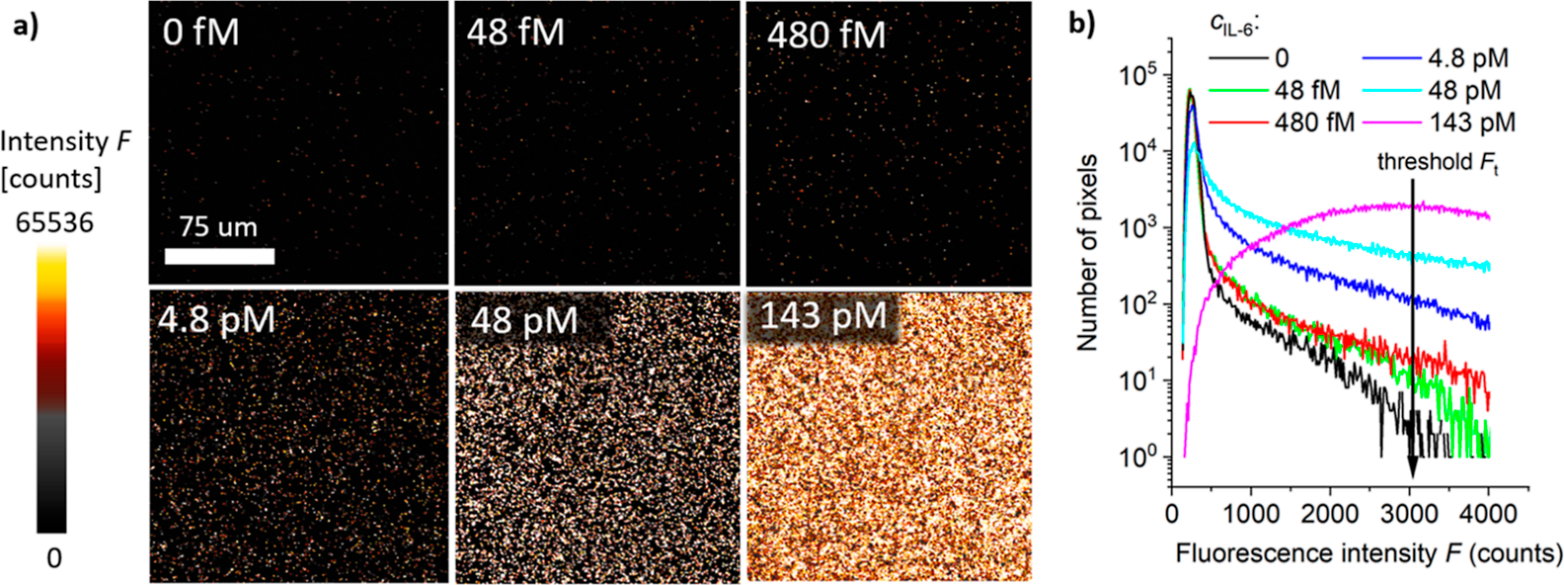
(a) Fluorescence images of a sensor chip surface after RCA sandwich immunoassay for target analyte concentration *c* = 0, 48 fM, 0.48 pM, 4.8 pM, 48 pM, and 143 pM followed by labeling with LS-Cy5. (b) Respective histograms of the fluorescence intensity. SAM biointerface architecture with cAb-biotin anchored via NA and dAb-CS* conjugated by maleimide chemistry. dAb-CS* dissolved in Tris pH = 8.4 and target analyte spiked in PBST.

After the experiment, the sensor surface of the reaction channels was contacted with PBST and imaged with the confocal fluorescence microscope. As shown in Figure 6a, the presence of RCA product manifests itself as increased fluorescence intensity originating from Cy5-LS labeled ssDNA chains attached to affinity captured IL-6 analyte. The imaging revealed spatially distinct bright spots that can be attributed to individual amplification events when target IL-6 molecules are captured from a solution with a low (fM and low pM) concentration *c*. For higher IL-6 concentration *c* > 48 pM the surface accommodates bright areas that are not separated. It should be noted that the ability of imaging the presence of individual IL-6 molecules on the sensor surface does not mean that the reported concept allows for the detection of individual copies of the analyte in the analyzed solution. Due to the restricted area that is used for the imaging and the diffusion -limited mass transfer from the bulk solution to the surface, it is expected that only a small fraction of molecules is captured and eventually detected. In addition, the assay was tested with shortened RCA reaction time *t* = 15, 30, and 45 min using the concentration of the analyte *c* = 47.6 pM (see Figure S6). All experiments showed a measurable average fluorescence intensity *F*_a_ indicating a possible shortening of the assay time.

In order to define the sensor output in terms of the number of spots that represent the individual captured analyte molecules, a fluorescence intensity threshold *F*_t_ was defined. The threshold *F*_t_ was set based on the plotted histograms of the fluorescence intensity for changing concentration of target analyte *c*. As seen in Figure 6b, the threshold *F*_t_ is positioned at the level where the fluorescence intensity occurrence changes by the highest magnitude with the analyte concentration *c*.

**Figure 7:**
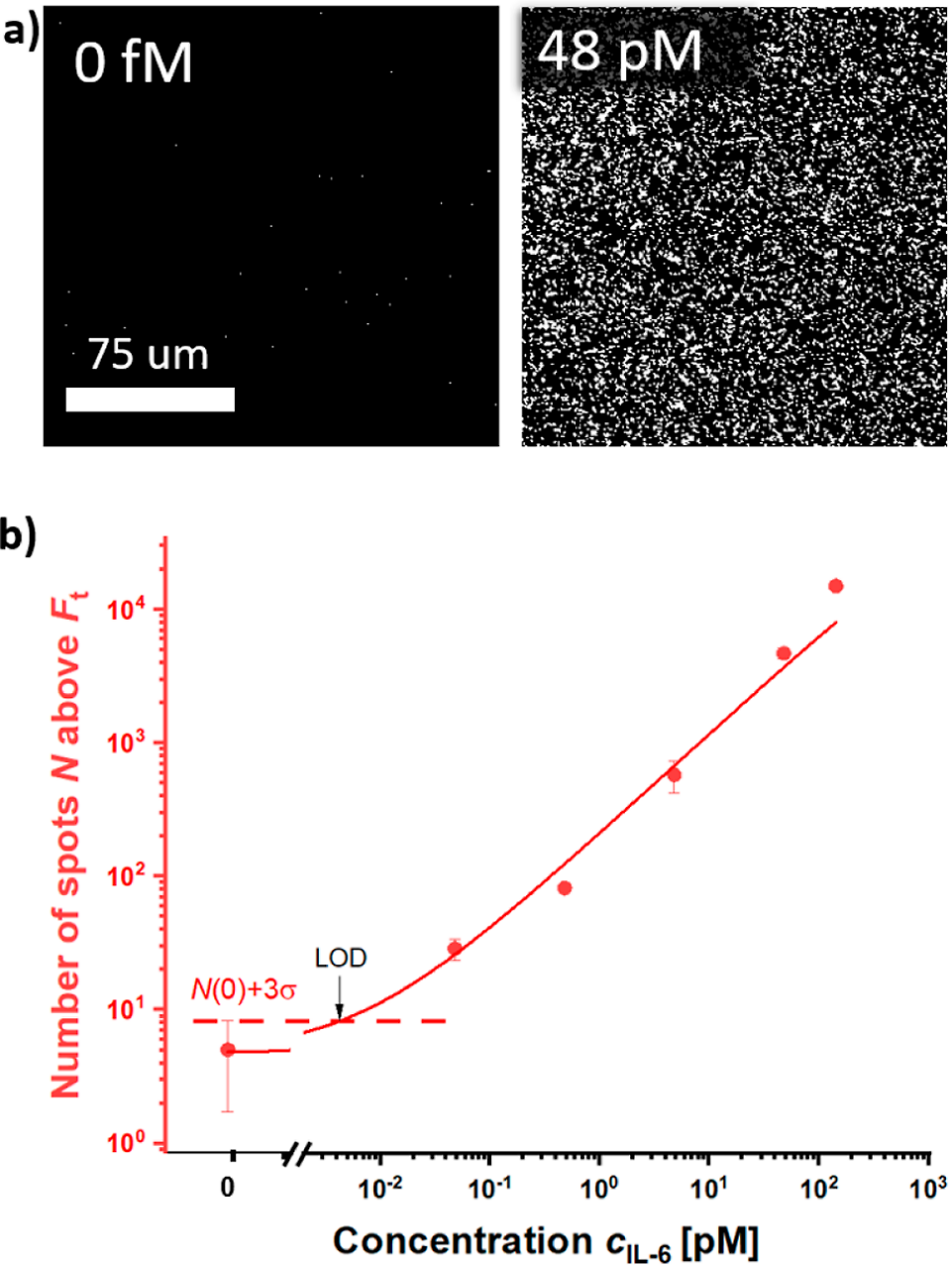
(a) Fluorescence images showing pixels above the defined threshold *F*_t_ = 3000 counts for target analyte concentration *c* = 0, 48 fM, and 48 pM. from Figure 6. (b) Established calibration curve based on counting of pixels above the threshold.

Figure 7a shows an example of the acquired image where the pixels above the determined threshold *F*_t_ = 3000 are visualized for target analyte concentration of *c* = 0 and 48 pM. The number of spots *N* above this threshold were then used to establish a calibration curve of a “digital” readout format of the assay, as presented in Figure 7b. The LOD was then determined from the control experiment performed with a blank sample, yielding a value of 4.3 fM.

### Validation for Analysis of Human Serum Samples

In the last step, the proposed assay concept was tested for the analysis of target analyte present in human blood serum, where other abundant interfering molecules may hinder analytical performance. The platform was tested for detection of IL-6 in standardized serum samples containing a full matrix of multiple proteins, including growth factors, cytokines, and other signaling molecules. There were employed standards with fM to low pM IL-6 concentrations with a 10-fold dilution with a working buffer prior to analysis by the immuno-RCA assay. As control, the reference channel of the microfluidic device was modified with biotin-BSA instead of the biotin-cAb that were immobilized in the measuring channels. For specificity testing of the IL-6 assay, such a control experiment was also conducted with biotin-anti-TNF-alpha antibody, as shown in Figure S8. The RCA process was conducted for a decreased time of 30 min since it was proved to generate sufficiently strong optical signal with bright spots identifying the presence of affinity captured IL-6 molecules.

**Figure 8:**
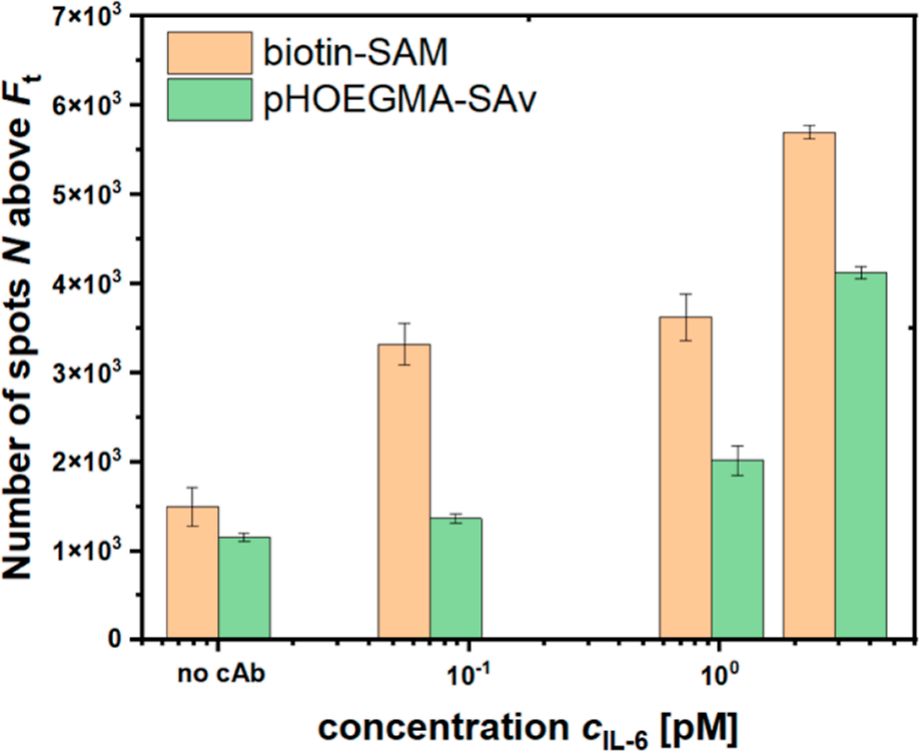
Comparison of calibration curves measured for IL-6 detection of human serum samples with IL-6 diluted 1:10 with PBST on the biointerface with cAb-biotin anchored by using SAM and pHOEGMA polymer brush.

The detection was performed in triplets, accommodating the control and the serum samples with *c* = 2.89 pM, 930 fM, and 70 fM on one sensor chip. After the surface was imaged with the confocal fluorescence microscope, the threshold *F*_t_ was set for each experiment according to the procedure described for the spiked buffer samples before. Respective histograms of all three experiments are shown in Figure S7a–c. In Figure 8, the acquired results are presented for the threshold of *F*_t_ = 1000 and error bars are determined from values obtained at different locations on the surface. These data show that the developed assay allowed the presence of IL-6 to be distinguished in all tested samples with a response significantly above *N* acquired from the control channel. For the pHOEGMA surface, the response *N* scales with analyte concentration *c*, while for the simpler biotin-SAM the acquired response led to less reproducible outcome not suitable for reliable analysis.

## Conclusions

We report on the successful implementation of single protein molecule detection via an enzymatic amplification technique based on immuno-RCA in a sandwich format. For ultrasensitive detection requiring minimized background that masks the specific sensor signal, tailoring of the biointerface is reported by addressing the interaction between the assay constituents and the biointerface. At optimized conditions using a charge neutral pHOEGMA polymer brush biointerface and weak Coulombic repelling between the surface-attached capture antibody and detection antibody tagged with short ssDNA chains present in the solution, the visualization of individual target analyte molecules that were affinity captured on the surface was possible by fluorescence microscopy. This allowed for setting up a readout resembling digital assays when individual molecules are counted (compared with the traditional detection when the output signal is averaged over the ensemble of target molecular species). The presented biosensor was demonstrated to detect IL-6 analyte at a low fM concentration, and it can be adapted to other relevant biomarkers. Since it is based on a combination of affinity capture of the target species on a solid surface in conjunction with a microfluidic device for sample and reagent delivery, it offers a straightforward route for future multiplexed detection. Through the minimizing of false positive response, the reported implementation of immuno-RCA can be used in clinical settings for complex biological samples such as human serum. In comparison to other immuno-RCA assays (summarized in Table S1) the reported approach advanced by antifouling biointerface shows comparable LOD to the best reported works and it holds potential to simplify ultrasensitive analysis for multiplexed detection of proteins. In addition, it allows us to implement SMD without the need of compartmenting of the sample that is used in the already established digital ELISA method.^26^

## Supporting Information Available

Schematics of chemical structures describing the preparation of streptavidin-modified pHOEGMA brushes and the conjugation of the detection antibody to the DNA primer sequence, agarose gel-electrophoresis result, additional data for SPR/SPFS measurements on polymer brushes, fluorescence microscopy data for time-dependence of RCA reaction timing, analysis of human serum samples and specificity testing, and overview of reported immuno-RCA assays (PDF)
