# Supplementary material for: Sandwich Immuno-RCA Assay with Single Molecule Counting Readout: The Importance of Biointerface Design
Source: ACS Appl Mater Interfaces. 2024 Mar 26;16(14):17109–19. doi: 10.1021/acsami.3c18304 (PMC11009916; doi:10.1021/acsami.3c18304)
Supplement: Supplementary file 1 — am3c18304_si_001.pdf [file am3c18304_si_001.pdf]

Supporting Information to:

Sandwich immuno-RCA assay with single molecule counting readout: the importance of biointerface design

Katharina Schmidt†, Tomas Riedel°, Andres de los Santos Pereira°, N. Scott Lynn Jr.⊥, Diego Fernando Dorado Daza°, Jakub Dostalek†⊥

† Laboratory for Life Sciences and Technology (LiST), Danube Private University, Viktor-Kaplan-Straße 2, 2700 Wiener Neustadt, Austria

° Institute of Macromolecular Chemistry, Czech Academy of Sciences, Heyrovského nám. 2, Prague 162 00, Czech Republic

⊥ FZU-Institute of Physics, Czech Academy of Sciences, Na Slovance 2, Prague 182 21, Czech Republic

Corresponding Author

*E-mail: jakub.dostalek@dpu-uni.at.

Table of content:

1. Preparation of pHOEGMA-SAv brushes
2. Conjugation of detection antibody to primer sequence CS*
3. RCA reaction characterization by agarose gel-electrophoresis
4. SPR/PEF measurement on pHOEGMA-SAv brushes
5. Immuno-RCA assay on poly(HPMA-*co*-CBMA) brushes
6. Dependence on RCA-reaction time
7. Serum samples
8. Specificity testing of biosensor
9. Overview for immuno-RCA assays

1. Preparation of pHOEGMA-SAv brushes

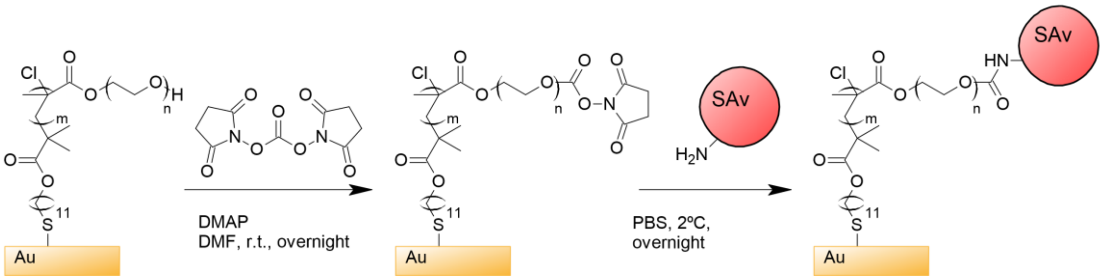

Figure S1. Scheme of the functionalization of pHOEGMA brushes with SAv (DMAP: 4-dimethylaminopyridine, DMF: dimethylformamide).

2. Conjugation of detection antibody to primer sequence CS*

The conjugation of biotin-dAb was performed in an *in-situ* step on the cAb modified carboxy-SAM by flowing streptavidin over the surface with four binding pockets, allowing the biotinylated primer sequence to bind, see Figure S2a. The click chemistry was done in a similar way, however the dAb was pre-modified *ex-situ* with DBCO-NHS ester, which could click to the azide tagged CS* *in-situ* (Figure S2b). As shown in Figure 3c, the maleimide-NHS ester was attached to the amine groups of the dAb and in a subsequent *ex-situ* step coupled to the sulfhydryl-activated CS* to form the dAb-CS* complex for the experiments on the biotin-SAM.

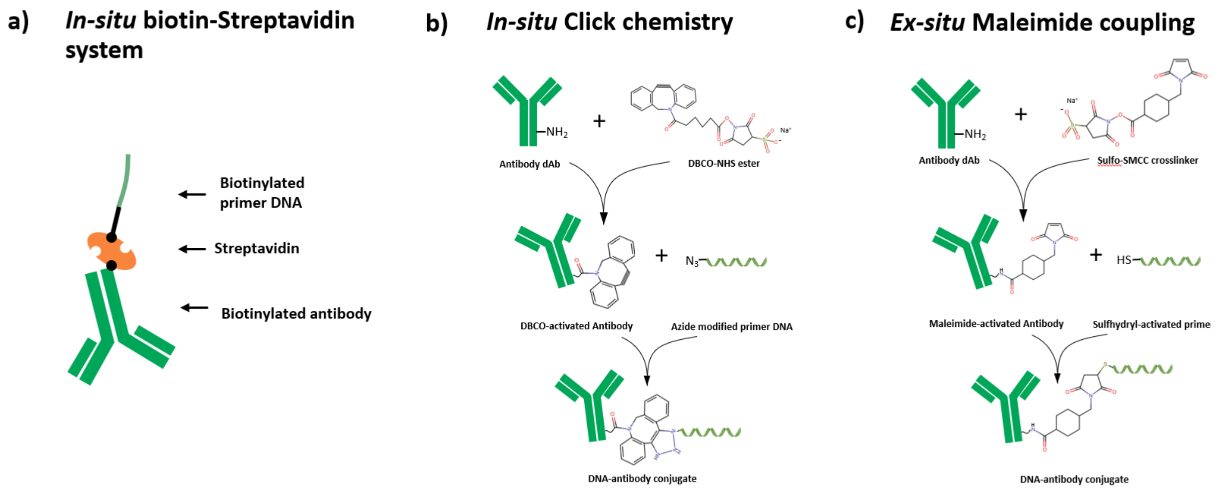

Figure S2. Schematical drawings for the conjugation of dAb to CS* via a) *in-situ* biotin and streptavidin bridge, b) *in-situ* click chemistry and c) *ex-situ* maleimide coupling.

3. RCA reaction characterization by agarose gel-electrophoresis

The RCA reaction was performed in solution for 10 min, 20 min, 30 min and 1 h and afterwards loaded on an 0.8%-agarose gel for separation and visualization of the RCA generated DNA product. The amplification reaction was conducted with 20 µL of PL with molar concentration c = 40 nM, 2 µL of biotin/20T/TS* (40 nM), 1 µL of dNTPs (25 µM), ϕ29-Polymerase (20 units) and 13 µL NFW-BSA (0.2 mg/mL) on the HulaMixer at room temperature. After incubation of the indicated time, the reaction was stopped by inactivation of the enzyme on the thermomixer at 70°C and 700 rpm for 10 min. Then the DNA was loaded with 1:10 diluted loading dye into the pockets of 50 µL of the agarose gel for size separation at 100 V for 30 to 40 min.

Figure S3 shows the imaged gel with dark bands indicating the presence of the loaded DNA. Lane 1 and 2 show the 1 kilobasepair (kb) and 100 basepair (bp) ladder. However, after 10 min the RCA product already reaches a length, which can barely escape the loading pocket. With increasing RCA time this effect is even more pronounced. Lane 3 to 5 show the PL after ligation with molar concentrations of c = 40 nM, 4 nM and 0.4 nM in which the latter does not show a band due to low concentration.

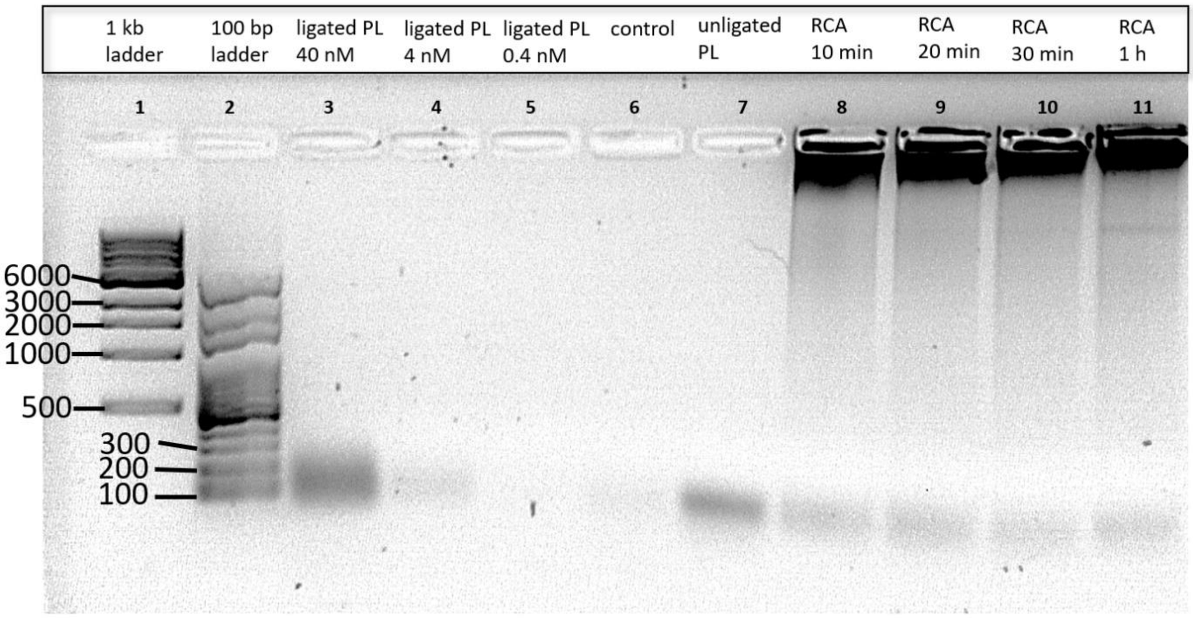

Figure S3. Agarose gel-electrophoresis: lane 1: 1 kb ladder; lane 2: 100 bp ladder; lane 3 to 5: ligated PL with c = 40 nM, 4 nM and 0.4 nM; lane 6: control sample; lane 7: unligated PL with c =40 nM and lane 8 to 11: RCA reaction conducted for 10 min, 20 min, 30 min and 1 h.

4. SPR/PEF measurement on pHOEGMA-SAv brushes

The SA modified pHOEGMA-brushes were swollen in PBST and after 100 min coupled to biotin-cAb for the detection of IL-6 with molar concentration c = 48 nM. The maleimide coupled dAb-CS* complex was diluted in Tris-HCl buffer (pH 8.4) to which the PL could hybridize for the subsequent start of the RCA process for 60 min, monitored over time, shown in Figure S3a. Afterwards the generated ssDNA was labeled with Cy5-LS, producing a strong fluorescence signal $\Delta F = 1.0 \cdot 10^5$ cps, which is enhanced 1.7 and 2.7 times when contacting the surface with molar concentrations of $CaCl_2$ c = 10 mM and 100 mM, as shown in the angular reflectivity $R(\theta)$ and fluorescence scans $F(\theta)$ in Figure S3b.

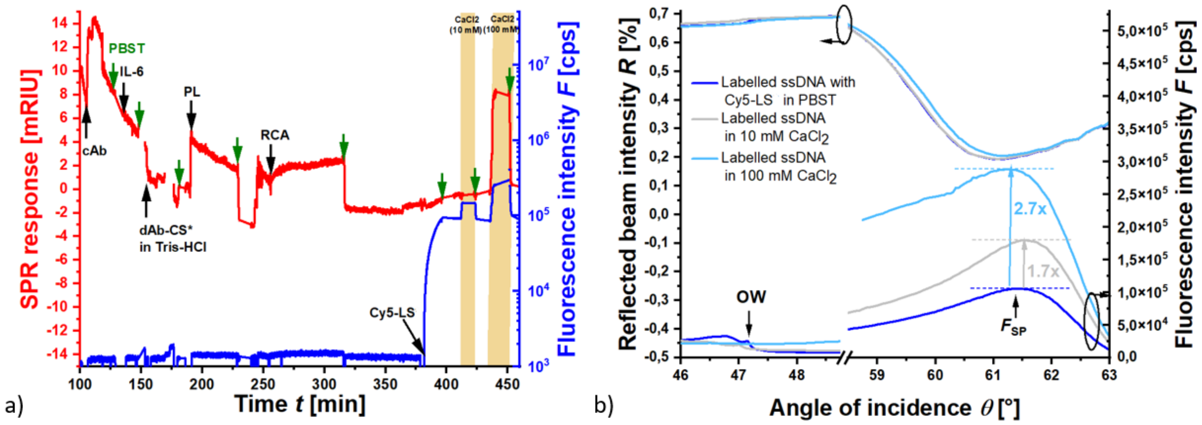

Figure S4. a) $R(t)$ in red and $F(t)$ in blue of immuno-RCA on pHOEGMA-SAv brushes with c_{IL-6} = 48 nM and b) respective angular reflectivity $R(\theta)$ and fluorescence scans $F(\theta)$.

5. Immuno-RCA assay on poly(HPMA-*co*-CBMA) brushes

The averaged fluorescence intensity plotted in Figure S4 was deduced from the images acquired by the confocal fluorescence microscope. The carboxy groups of the poly(HPMA-*co*-CBMA) brushes were prepared according to a previously published procedure and functionalized via the EDC/NHS coupling with cAb specific to IL-6. However, the surface was not contacted with any analyte, but tested for the unspecific response. To reduce the fluorescence signal originating from binding of assay constituents by active groups or the disruption of the neutral charge balance, the following deactivation agents with either sulfate/sulfo groups or carboxy groups were tested to deactivate the residual NHS esters on the surface: Tris-HCl (10 mM) with pH = 8.4, glycine (1 M) with pH = 7, spermine (20 mM) in HEPES buffer with pH = 8 and different ratios of glycine (G) to 2-aminoethyl hydrogen sulfate (D1) and aminomethanesulfonic acid (D2), see Figure S4a. Additionally, changing of the dilution buffer of the dAb-CS* complex for the deactivated surface with 96G:4D1 was tested (Figure S4b).

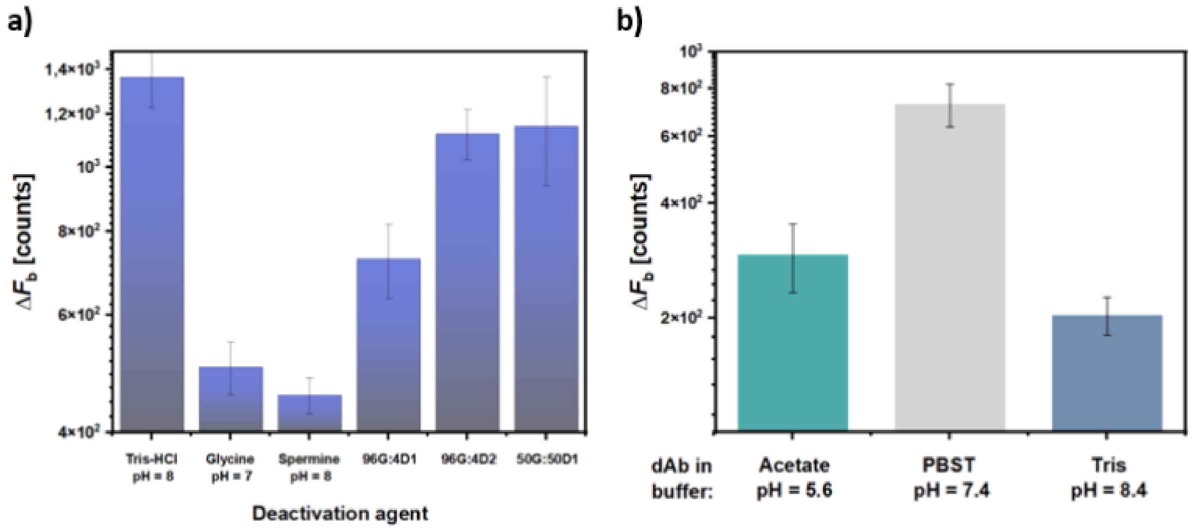

Figure S5. Plotted average fluorescence intensity acquired from experiments with immuno-RCA with running time of 60 min for IL-6 with *c* = 0 pM on poly(HPMA-*co*-CBMA) brushes testing a) different deactivation agents and b) buffer for dAb-CS* conjugate after deactivating residual NHS ester with 96G:4D1

6. Dependence on RCA reaction time

Experiments have been performed according to the description in the 'Methods' section with the four-channel microfluidics mounted on the gold slide modified with the biotin-SAM. The analyte concentration was kept constant at c = 48 pM and the maleimide coupled dAb-CS* was diluted in Tris-HCl buffer with pH = 8.4. After the hybridization event of the PL, the RCA was conducted for 15 min, 30 min, 45 min and 60 min, respectively. For the control experiments for the same RCA timings, the surface was not in contact with the analyte. As depicted in Figure S5a, the average fluorescence intensity gradually increased with longer RCA running times. Even the image after 15 min of RCA time showed significantly higher fluorescence signal F than the image of the control channel (see Figure 5b).

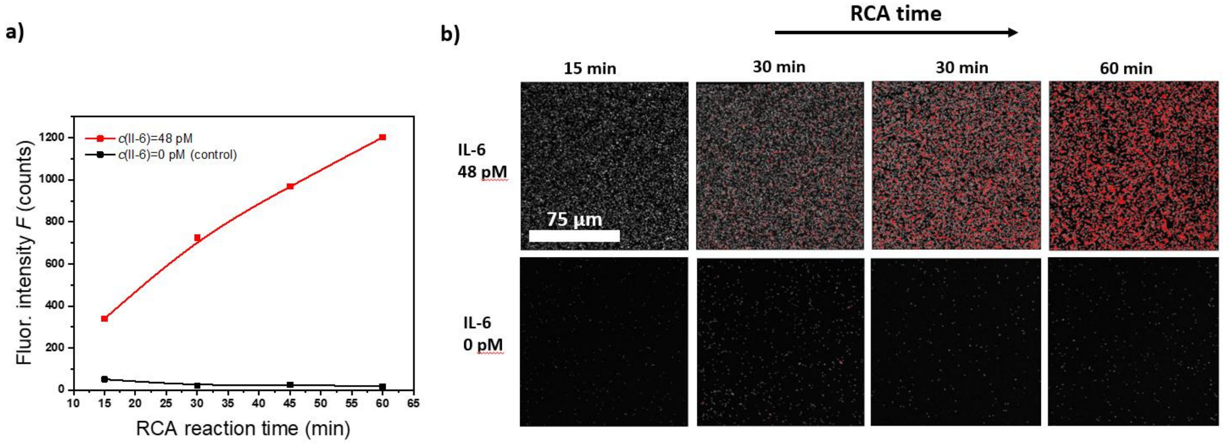

Figure S6. a) Plotted average fluorescence intensity acquired from experiments with RCA running time of 15 min, 30 min, 45 min and 60 min with c = 48 pM in red and c = 0 pM of IL-6 in black and b) the respective images from the confocal fluorescence microscope.

7. Serum samples

The experiments were performed as triplets on the pHOEGMA-SAv brushes with three different molar concentrations of IL-6 c = 2.89 pM, 930 fM and 70 fM and a control channel with c = 0 pM on one sensor slide with the microfluidic system and imaged after running the RCA for 30 min with the confocal fluorescence microscope. In Figure S6, the histograms of all three experiments are plotted, for which the threshold has to be determined individually.

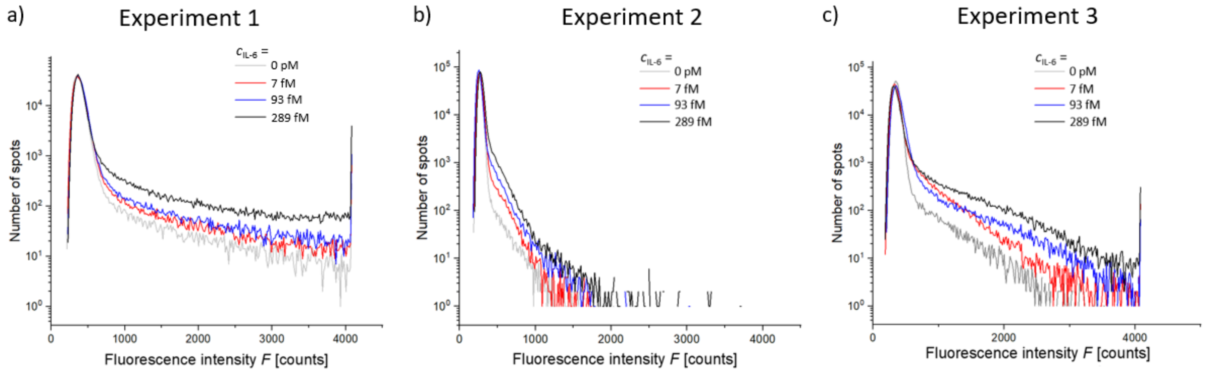

Figure S7. a) – c) Plotted respective histograms of images acquired from the areas contacted with molar concentration of IL-6 of c = 2.89 pM, 930 fM, 70 fM and 0 pM from three different experiments.

8. Specificity testing of biosensor

Experiments were conducted in the 4-channel microfluidic system with fluorescence microscopy read-out for counting of number of spots N. As control of the specific capture of IL-6 from 1:10 diluted serum samples, the biotin-SAM surface was modified with either biotin-anti-TNF-alpha or biotin-BSA coupled via the neutravidin layer instead of the specific cAb to IL-6. After flowing the serum samples containing multiple potentially interfering biomolecules such as vascular endothelial growth factor, tumor necrosis factor alpha, epidermal growth factor, various interleukins, monocyte chemoattractant protein-1 and interferon gamma over the surface, the dAb-CS* dissolved in Tris-HCl (pH = 8.4) buffer and the PL were conducted according to previous experiments and the RCA was run for 30 min. Figure S8 shows the ratio of specific N_a acquired from the serum samples with molar concentration of IL-6 c = 2.89 pM, 930 fM and 70 fM to unspecific response N_b with molar concentration of IL-6 c = 0 pM after setting the threshold to F_t = 1000 and counting of particles with ImageJ.

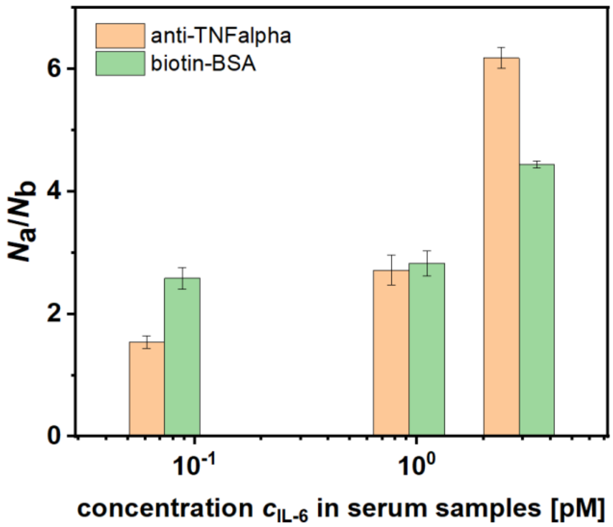

Figure S8. Specificity testing of immuno-RCA assay for IL-6 detection with serum samples of c = 2.89 pM, 930 fM and 70 fM on biotin-SAM biointerface modified with either anti-TNF-alpha or biotin-BSA as cAb.

9. Overview for immuno-RCA assays

RCA already served as amplification technique in conjunction with immuno-assays for detection of different biomarkers. Table S1 provides an overview of the assays reported in literature and the stated analytical performance (limit of detection).

Table S1. Examples for immuno-RCA assays and the reported limit of detection for the specific analyte.

Detection method	Analyte	Amplification strategy	Biointerface	Tested in real samples	Limit of detection	SMD	Reference
Chemiluminescence	human prolactin	RCA with streptavidin-horseradish peroxidases	microplate well	serum	0.01 fM	No	http://dx.doi.org/10.1016/j.snb.2015.06.063
Electrochemical	human vascular endothelial growth factor	RCA with quantum dots	microplate well	Cell culture fluid	10 fM	No	10.1021/ac1001449
Fluorescence	prostate specific antigen, carcinoembryonic antigen, a-fetoprotein	RCA with suspension bead array	polystyrene beads modified with carboxy-groups	serum	0.1 pM, 50 fM, 9 fM	No	https://doi.org/10.1016/j.aca.2018.10.001
Fluorescence	anti-ADAMTS13	RCA	glass substrate modified with aldehyde-groups	plasma	0.63 pM	No	https://doi.org/10.1021/acs.analchem.1c00172
Fluorescence	prostate specific antigen	RCA	glass substrate modified with thiol-silane	serum	2.9 fM	Yes	10.1073ypnas.170237197.
Fluorescence	IgG	RCA with fluorescent DNA Nanotags	glass substrate modified with epoxy-groups	Serum, cell culture fluid	0.9 fM	No	dx.doi.org/10.1021/bc200537g
SPR	human vascular endothelial growth factor	RCA	polystyrene microspheres modified with carboxy-groups	No	2.22 pM	No	http://dx.doi.org/10.1016/j.bios.2014.05.005
